# Genome Sequences of Extended-Spectrum Beta-Lactamase-Producing *Escherichia coli* Recovered from Mid-Stream Urine Samples in Accra, Ghana

**DOI:** 10.3390/microorganisms12061139

**Published:** 2024-06-04

**Authors:** Nicholas T. K. D. Dayie, Beverly Egyir, Felicia Amoa-Owusu, Christian Owusu-Nyantakyi, Bright Adu, Fleischer C. N. Kotey, Eric S. Donkor, Richard A. Stabler

**Affiliations:** 1 Department of Medical Microbiology, University of Ghana Medical School, Accra P.O. Box KB 4236, Ghana; ntkddayie@ug.edu.gh (N.T.K.D.D.); fleischarles@gmail.com (F.C.N.K.); esampane-donkor@ug.edu.gh (E.S.D.); 2 Department of Bacteriology, Noguchi Memorial Institute for Medical Research, University of Ghana, Accra P.O. Box LG 581, Ghana; begyir@noguchi.ug.edu.gh (B.E.); owusufelicia90@gmail.com (F.A.-O.); cowusu-nyantakyi@noguchi.ug.edu.gh (C.O.-N.); 3 Department of Immunology, Noguchi Memorial Institute for Medical Research, University of Ghana, Accra P.O. Box LG 581, Ghana; badu@noguchi.ug.edu.gh; 4 Department of Infection Biology, London School of Hygiene and Tropical Medicine, London WC1E 7HT, UK

**Keywords:** *Escherichia coli*, extended-spectrum beta-lactamase, ESBL, Ghana

## Abstract

*Escherichia coli*, a member of the commensal intestinal microbiota, is a significant aetiology of urinary tract infections (UTIs) and has a propensity for acquiring multidrug resistance characteristics, such as extended-spectrum beta-lactamases (ESBLs). Despite the increase in the incidence of ESBL-producing *E. coli* infections in sub-Saharan Africa, routine ESBL detection in Ghana is often absent, and molecular data on ESBL genotypes is scarce. Eleven ESBL-producing *E. coli* recovered from mid-stream urine samples were subjected to antimicrobial susceptibility testing and whole-genome sequence analyses. All isolates exhibited multidrug resistance, demonstrating phenotypic resistance to third-generation cephalosporins, such as cefotaxime, ceftazidime, and cefpodoxime. Three isolates demonstrated resistance to norfloxacin (a fluoroquinolone), and one isolate demonstrated intermediate resistance to ertapenem (a carbapenem). Analysis of the draft genomes identified multiple antimicrobial resistance genes including ESBL genotypes *bla*_TEM-1B/TEM-190_ (6/11 and 1/11, respectively), *bla*_CTX-M-15/CTX-M-3_ (7/11 and 1/11) and *bla*_OXA-1/OXA-181_ (3/11 and 1/11). The strains belong to 10 different serotypes and 10 different multilocus sequence types. This study provides information on phenotypic resistance in 11 ESBL *E. coli* from Ghana and AMR genotypes within their genomes.

## 1. Introduction

*Escherichia coli* is a natural member of the human microbiota and is a significant aetiology of urinary tract infections (UTIs) [1]. *E. coli* is able to exchange genetic material, including antibiotic resistance determinants, which may be plasmids or other mobile genetic elements, with other intestinal commensals [2]. One important group of antibiotic resistance determinants is beta-lactamases (encoded by *bla* genes), which hydrolyse the beta-lactam ring in penicillin and first- and second-generation cephalosporins (1GC and 2GC, respectively) [3,4,5,6,7].

Beta-lactamases are divided into the following four classes: the active site serine β-lactamases (Classes A, C, and D) and the zinc-dependent or metallo-β-lactamases (MBLs; Class B) [8]. Extended-spectrum beta-lactamases (ESBLs) are capable of hydrolysing antibiotics, such as aztreonam and fourth-generation cephalosporins (4GCs) [9]. Classification of the cephalosporins into first-, second-, third- (3GCs), fourth- (4GCs), and fifth-generation (5GCs) cephalosporins reflects a mixture of their temporal discovery and their spectrum of activity against Gram-negative and Gram-positive bacteria. Over 400 ESBL genes have been documented within Class A (*bla*_TEM_, *bla*_SHV_, and *bla*_CTX-M_) and Class D (*bla*_OXA_) β-lactamases. Class D (*bla*_OXA_)-encoded beta-lactamases are frequently encountered in *Klebsiella pneumoniae* and *E. coli*, mediating carbapenem (doripenem, meropenem, ertapenem, and imipenem) resistance [10,11].

There is an increase in the incidence of infections caused by ESBL-producing *E. coli* globally and in sub-Saharan Africa [12,13]. Previous studies in Ghana have indicated high positivity rates (49.3–61.1%) for ESBLs among *E. coli* isolates recovered from clinical sources [14]. This is of public health concern, particularly because ESBL detection among Gram-negative bacterial species is not routinely performed in the majority of the clinical microbiology laboratories in Ghana. In addition, very little is known about the antimicrobial resistance and virulence gene content of ESBLs in the country, leaving noticeable gaps in antimicrobial resistance surveillance data [15]. This study, therefore, characterised 11 ESBL-producing *E. coli* using whole-genome sequencing and antimicrobial susceptibility testing to augment existing knowledge.

## 2. Materials and Methods

### 2.1. Phenotypic Investigations

Eleven (11) archived *E. coli* isolates that were recovered from mid-stream urine samples (from two hospitals in Accra, Ghana, during laboratory-based surveillance conducted between April 2017 and March 2018) and were confirmed by colonial morphology, Gram staining and Matrix-assisted Laser Desorption/Ionisation Time of Flight (MALDI-TOF) Mass Spectrometry (Bruker Daltonics, Bremen, Germany) using Biotyper™ system 2.0 software at the genus [log(score) 1.7–2.0)] and species [log(score) ≥ 2.0] levels. Antimicrobial susceptibility testing was performed using the Kirby-Bauer disc diffusion method and interpreted following the Clinical and Laboratory Standards Institute (CLSI) guidelines [16]. *E. coli* ATCC 25922 and *K. pneumoniae* ATCC 700603 were used as control strains. ESBL was confirmed by the double-disc method with cefotaxime, ceftazidime, and cefepime, alone and in combination with clavulanic acid [17]. Inhibition zone differences of ≥5 mm between the single and the combination discs for cefotaxime, ceftazidime, or cefepime were regarded as positive for ESBL production.

### 2.2. Whole-Genome Sequence Analysis of the Isolates

Whole-genome sequencing was performed at the Noguchi Memorial Institute for Medical Research, University of Ghana, on the eleven *E. coli* isolates. Genomic DNA was extracted using a standard genomic DNA extraction kit (Qiagen, Hilden, Germany). Extracted DNA was quantified using a Qubit 3.0^®^ Fluorometer with a Qubit high-sensitivity kit (Thermo Fisher Scientific, Toh Tuck, Singapore). Genomic libraries were prepared using the Nextera DNA flex library preparation kit as per the manufacturer’s instructions. Sequencing libraries were quantified using the 2100 bioanalyser system (Agilent, Santa Clara, CA, USA) and Kapa Sybr Fast qPCR kit (Kapa Biosystems, Wilmington, MA, USA). Libraries were sequenced using the MiSeq sequencer (Illumina, San Diego, CA, USA). Raw fastq files were quality-filtered to Phred score ≥20, filtered for a minimum read length of 50 bp, and adaptor-trimmed using BBDuk (BBMap, https://sourceforge.net/projects/bbmap/). The resultant high-quality reads were used for de novo assembly, employing the default settings of SPAdes assembler v 3.13.0. Draft genome multi-fasta files were evaluated using the Quast assessment tool v5.1.0rc1 [18]. Contigs were ordered against a UTI reference *E. coli* UTI89 (accession CP000432) using ABACAS v1.3.1 [19]. The resulting assemblies were polished using Pilon v1.22 [20] and annotated using Prokka v1.14.6 [21]. MLST (Achtman scheme) was predicted using MLST (Seemann T, mlst, Github https://github.com/tseemann/mlst, v2.19.0, database updated on 1 October 2020) and serotypes using SerotypeFinder 2.0 [22] and abricate --db ecOH (Seemann T, Abricate, Github https://github.com/tseemann/abricate, v1.0.0, database updated on 1 October 2020). AMR genes were identified using Abricate with the ‘CARD’ and ‘ResFinder’ databases, with a minimum identity of 90% and a minimum coverage of 90%. Plasmids were identified using Abricate with the ‘PlasmidFinder’ database with a minimum identity of 80% and a minimum coverage of 90%. Heatmaps and hierarchical clustering were generated using Morpheus (https://software.broadinstitute.org/morpheus/, accessed on 1 October 2021). The data sets presented in this study can be found in the online repository https://www.ncbi.nlm.nih.gov/bioproject/767840 as follows: isolate 0190 (accession number JAIZOE000000000), 0232 (JAIZOC000000000), 0320 (JAIZOB000000000), 0456 (JAIZOA000000000), 0513 (JAIZNZ000000000), 0533 (JAIZNY000000000), 0549 (JAIZNX000000000), 0198 (JAIZOD000000000), 0135 (JAIZNW000000000), 0350 (JAIZNV000000000), and 0492 (JAIZNU000000000).

## 3. Results

### 3.1. Antimicrobial Resistance and Serotypes of the Isolates

Antimicrobial susceptibility testing (AST) demonstrated high levels of resistance to β-lactam antibiotics. Only Isolate 0350 was susceptible to ampicillin, cefuroxime (2GCs), and ceftriaxone (a 3GC), and only Isolates 0232 and 0320 were susceptible to ceftazidime (a 3GC) (Table 1). All eleven isolates were resistant to cefotaxime (a 3GC) and cefpodoxime (a 3GC) (Table 1). Three isolates (27%) (0198, 0232, and 0456) were resistant to norfloxacin (a fluoroquinolone), and no isolate was resistant to ertapenem, although Isolate 0232 was classed as intermediate resistant on retesting by Microscan (Renton, WA, USA) (Table 1).

Draft genomes were assembled for each isolate (Table 2), which consisted of an average of 156 contigs (84–271 contigs, SD ± 54), 5,034,761 bp (5,339,158–4,655,431 bp, SD ± 204), 220 bp, and 4696 CDSs (4380–4960, SD ± 202) (Table 2). All the isolates had evidence of plasmids through the presence of plasmid replicon sequences (Supplementary Table S4). The most common plasmid detected was the Col-like small plasmid, which was often also accompanied by an IncFI or IncFII incompatibility group plasmid. Interestingly, both Isolates 0513 and 0533 had replicons from both IncFI and IncFII incompatibility group plasmids (Supplementary Table S4). The genomic data were used to identify 10 different MLSTs (Achtman scheme). Isolates 0513 and 0533 were both ST131 strains (Table 1). Isolate 0190 had a novel *gyrB* allele along with *adk*(6), *fumC*(4), *icd*(18), *mdh*(9), *purA*(26), and *recA*(7), which are found in STs 99, 1611, 2063, 6401, 10050, and 11138. Three isolates (0198, 0320, and 0135) were from the same MLST clonal complex (CC), CC10 (Table 1), but three isolates (0190, 0456, and 0549) were sporadic and not associated with any specific complex (Table 1).

Serotypes were predicted from the assembly data and were unique to each isolate, with the exception of Isolates 0513 and 0533 (Table 1). SerotypeFinder did not identify an O type for Isolate 0456, although it was predicted to be H8:O8 by Abricate. It also gave an ambiguous O type to 0320 because of the similarity to multiple genes; O62 *wzm* (100% identity), O68 *wzx* (99.92%), and O68 *wzy* (99.91%) (Supplementary Table S1). Analysis using Abrogate identified 0320 as O62:H30 (Supplementary Table S1).

### 3.2. Overview of the Identified Genomes

**Figure 1:**
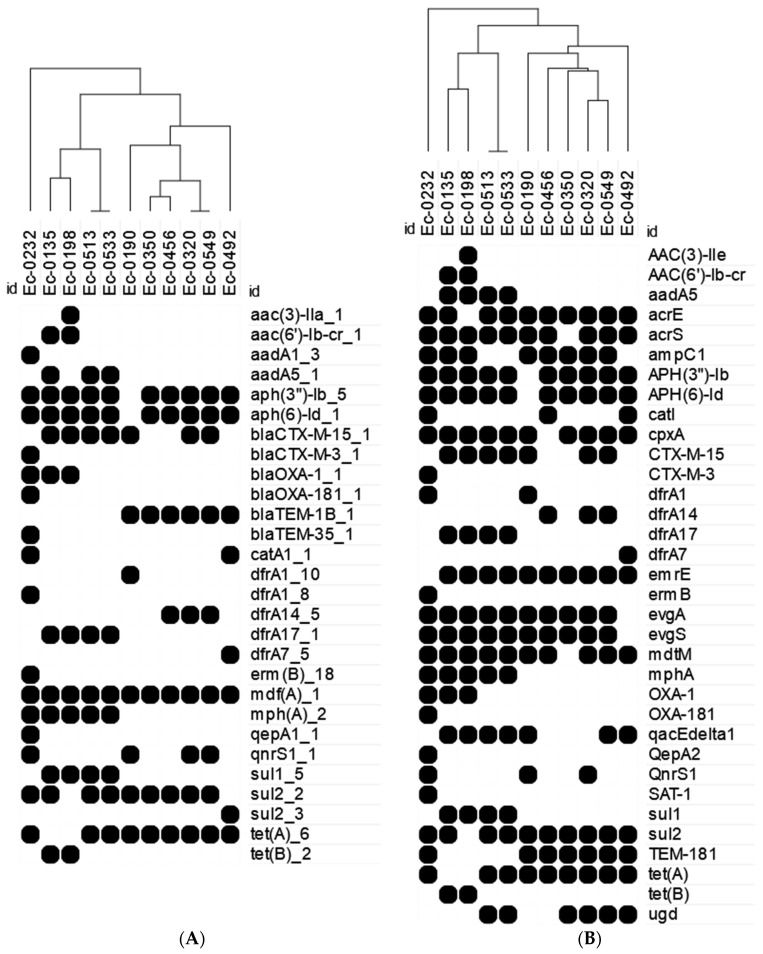
Presence of genes linked to AMR within the *E. coli* isolates. Black circles indicate that a gene was identified by Abricate using CARD databases within a draft genome. Isolates were clustered using the Euclidian distance algorithm. (**A**) Resfinder database. (**B**) CARD database; in addition, the following genes were conserved in all the 11 isolates: *acrA*, *acrB*, *acrD*, *acrF*, *ampC*, *ampH*, *bacA*, *baeR*, *baeS*, *CRP*, *emrA*, *emrB*, *emrK*, *emrR*, *emrY*, *eptA*, *gadW*, *gadX*, *H-NS*, *kdpE*, *marA*, *mdfA*, *mdtA*, *mdtB*, *mdtC*, *mdtE*, *mdtF*, *mdtG*, *mdtH*, *mdtN*, *mdtO*, *mdtP*, *msbA*, *pmrF*, *tolC*, and *yojI*.

Genome analysis for genes linked to antibiotic resistance against two curated databases, ResFinder and CARD, identified 28 and 70 genes, respectively (Figure 1A and Figure 1B, respectively). β-lactamase genes from Class A (*bla*_TEM_, and *bla*_CTX-M_) and Class D (*bla*_OXA_) were identified; *bla*_CTX-M-15_ was identified in 7/11 isolates, *bla*_CTX-M-3_ was identified in an eighth isolate (0232). Additionally, *bla*_TEM_ was present in 7/11 isolates—while CARD labeled all these as *bla*_TEM-181_ (Figure 1B), ResFinder distinguished the *bla*_TEM_ of Isolate 0232 as *bla*_TEM-35_, but those of the other six isolates as *bla*_TEM-1B_ (Figure 1A). Protein sequence analysis identified *bla*_TEM-35_ in Isolate 0232 and *bla*_TEM-1B_ in Isolates 0190, 0350, 0456, 0320, 0549, and 0492 (Figure 1A); *bla*_TEM-1B_ differs from *bla*_TEM-181_ because of an A182V protein substitution. The *bla*_OXA-1_ gene was present in three isolates (0232, 0135, and 0198); however, Isolate 0232 had, in addition, *bla*_OXA-181_ (Figure 1); *bla*_OXA-181_ belongs to the OXA-48-like family carbapenem-hydrolysing Class D β-lactamases. PlasmidFinder identified putative plasmids through the identification of replicon sequences; however, it was not possible to link the AMR genes with these plasmids.

Analysis of genotypic and phenotypic data revealed a wide diversity within the limited isolate collection with the exception of Isolates 0513 and 0533 (Table 1, Figure 1). Isolate 0513 and 0533 were both predicted to be ST131 and had the same AST profiles (Table 1). Additionally, the AMR genotypes identified were identical (Figure 1).

Isolate 0232 was an outlier from the other *E. coli* isolates when clustering by AMR genes and was predicted to contain six or seven CDSs (CARD and ResFinder, respectively, Supplementary Tables S2 and S3) that were not found in the other ten isolates (Figure 1A). Isolate 0232 was also the only strain to harbour two *bla*_OXA_ genes, and was also demonstrated to be intermediately resistant to ertapenem, but sensitive to ceftazidime (Table 1, Figure 1). Additionally, it was the only strain predicted to have *bla*_CTX-M-3_, which is only one amino acid different from *bla*_CTX-M-15_ (Asp240Gly); however, the difference in the omega loop region of *bla*_CTX-M-15_ results in increased ceftazidime hydrolysis and antibiotic resistance compared with *bla*_CTX-M-3_. Isolate 0320 was also identified to carry *bla*_CTM-X-15_ but was ceftazidime-sensitive.

Isolate 0350 was the only strain susceptible to ampicillin, cefuroxime, and ceftriaxone despite possessing *bla*_TEM-1B_, the most common plasmid-mediated β-lactamase of ampicillin resistance. The *bla*_TEM-1B_ protein sequence was identical in all six strains, with no mutations or frameshifts present in Isolate 0350. Genome analysis suggested that Isolate 0350 had only a few differences in AMR genes from its nearest and resistant neighbor (Figure 1); ResFinder only identified a lack of *drfA*14 compared with Isolate 0456, and CARD suggested Isolate 0350 was lacking *acrS*, *catI*, *dfrA14*, *mdtM*, and *ugd*, but contained a *cpxA* compared with Isolate 0456. These genes are not involved or targets for β-lactam resistance.

Three strains demonstrated resistance to norfloxacin (a fluroquinolone), which is primarily mediated through non-synonymous mutations in the DNA gyrase (*gyrA*/B) or topoisomerase (*parC*) genes. Analysis of the draft genomes demonstrated that compared with norfloxacin-sensitive isolates 0190, 0320, and 0513, resistant isolates 0198 and 0232 have a *GyrA* A83L and D87N mutation. Isolate 0198 also has an additional *GyrA* D78E mutation. No mutations were seen in the *GyrB* of resistant isolates compared to the sensitive consensus sequence. In *ParC*, Isolates 0198 and 0232 contained an S80I compared with the sensitive consensus sequence. Isolate 0465, which had phenotypic resistance to norfloxacin, did not have non-synonymous mutations within these genes compared to the sensitive strains.

## 4. Discussion

In this study, we expanded on the limited knowledge of ESBLs from Ghana. Resistance of isolates to ampicillin, cefuroxime, ceftriaxone, cefotaxime, cefpodoxime, and ceftazidime is consistent with the characteristics of ESBL-producing organisms. Moreover, high levels of resistance (88.9–100%) against ampicillin, cefuroxime, and cefotaxime have been reported among *E. coli* isolates in previous studies conducted in the country [23]. It is interesting that the proportion of ESBL-producing *E. coli* resistant to norfloxacin in the current study (3/11, 27%) is comparable to the pooled proportion (6/15, 40%) reported for ESBL-producing and non-producing *E. coli* isolated from urine samples in a previous study in Ghana [24]. No isolate was resistant to ertapenem despite the presence of carbapenem resistance determinants *bla*_OXA-1_ in three strains. A single isolate (0232) carried two *bla*_OXA_ penemases did show intermediate resistance to ertapenem, and the low level of resistance may be due to this antimicrobial being rarely used in Ghana. However, it is concerning that one strain demonstrated intermediate resistance to ertapenem despite its limited use clinically.

The *bla*_CTX-M_ and *bla*_TEM_ genes were common among the ESBL-producing *E. coli* isolates detected in the study by Donkor et al. [24]. Similarly, in the study by Deku et al. [25] involving a collection of *E. coli* isolates recovered from a variety of clinical specimens from Ho in Ghana—blood, high vaginal swab, pleural aspirates, sputum, ear swab, wound, and urine—*bla*_TEM-1_ and *bla*_CTX-M_ (*bla*_CTX-M-1_, *bla*_CTX-M-825_, and *bla*_CTX-M-914_) were the predominant genes detected among ESBL isolates. Likewise, in the study by Mahazu et al. [26], which was conducted at two major hospitals in Ghana and involved *E. coli* from a similar set of specimens, as well as urethral discharge, pus, ascitic fluid, semen, endocervical swabs, stool, and others, *bla*_CTX-M_ genes (*bla*_CTX-M-14_, *bla*_CTX-M-15_, and *bla*_CTX-M-27_) dominated. These may explain the high proportion of cefotaxime and ampicillin resistance in the current study. The *bla*_CTX-M-15_ gene and, to a relatively lesser extent, other *bla*_CTX-M_ variants, have also been reported to be prevalent in *E. coli* isolated from locally-bred and imported chicken in the country [27,28]. Hence, it seems that the *bla*_CTX-M_ genes may be widespread in the country, as confirmed in other previous reports [29,30], and could be harboured by non-typical urine *E. coli* pathotypes, such as the enteroinvasive, enteroaggregative, enteropathogenic, enteropathogenic, enterotoxigenic, and enterohaemorrhagic *E. coli*. Dissemination of AMR genes is often associated with plasmid transmission.

In the study by Mahazu et al. [26] involving *E. coli* isolated from multiple clinical specimens, the predominant sequence type was ST131 (25/102, 24.5%). Similarly, in a recent study in the country, ESBL-producing *E. coli* isolated from poultry predominantly belonged to ST10 (31/45, 69%) and, in humans, to ST131 (4/34, 12%) [12]. Other studies conducted in the African setting (Nigeria and Tanzania) have also commonly detected ST10 in poultry [31,32], while others conducted in European and Asian countries have additionally identified it in humans at high frequencies (9–11%) [33,34,35]. The detection of CC10 among ESBL-producing *E. coli* in the current study suggests a link to the livestock production chain. This is especially important as they could cyclically serve as sources of multidrug-resistant *E. coli* infections and reservoirs for the accumulation of multidrug-resistant *E. coli* and other Enterobacteriaceae shed by humans. CC131 is known to occur globally, is multidrug-resistant, commonly harbours the *bla*_CTX-M-15_ gene, and is associated with pandemics [36]. The two *E. coli* isolates that belonged to CC131 each harboured the *bla*_CTX-M-15_ gene and several other resistant genes, and could be of the same strain. Similarly, STs belonging to CC ST10 were also observed to possess several multidrug-resistant genes that may possibly explain the phenotypic resistances observed.

This study underscores the importance of enhancing antimicrobial stewardship programmes and utilising robust tools like whole-genome sequencing to generate detailed data for informing AMR surveillance efforts in the country. The high level of antimicrobial resistance could be a reflection of the prevalent overuse and misuse of antimicrobials in Ghana.

A limitation of this study was the few isolates from a single time point, which reduces the generalisability of the findings. Subsequent studies that evaluate a higher number of isolates could be helpful in validating the observations in this study.

## Figures and Tables

**Table 1:** Antibiotic susceptibility and clonal information of the ESBL *E. coli* isolates.

Isolate	AMP	CXM	CRO	CTX	CAZ	CFP	NOR	ETP	ST	CC	Predicted Serotype(s)
0190	R	R	R	R	R	R	S	S	– *	– *	O96:H19
0198	R	R	R	R	R	R	R	S	617	10	O101:H10
0232	R	R	R	R	S	R	R	I	205	205	O100:H12
0320	R	R	R	R	S	R	S	S	34	10	O62/O68:H30 [O62:H30]
0456	R	R	R	R	R	R	R	S	2522	–	H8 [O8:H8]
0513	R	R	R	R	R	R	S	S	131	131	O16:H5
0533	R	R	R	R	R	R	S	S	131	131	O16:H5
0549	R	R	R	R	R	R	S	S	1722	–	O1:H25
0492	R	R	R	R	R	R	S	S	12	12	O4:H1
0135	R	R	R	R	R	R	S	S	44	10	O101:H4
0350	S	S	S	R	R	R	S	S	69	69	O45:H16

AMP = ampicillin; CXM = cefuroxime (2nd-generation cephalosporin [2GC]); CRO = ceftriaxone (3rd generation cephalosporin [3GC]); CTX = cefotaxime (3GC); CAZ = ceftazidime (3GC); CFP = cefpodoxime (3GC), NOR = norfloxacin (fluoroquinolone); ETP = ertapenem (carbapenem). ST = multilocus sequence type; CC = clonal complex. Note, * 0190 had a novel variant of the *gyrB*(865) allele along with *adk*(6), *fumC*(4), *icd*(18), *mdh*(9), *purA*(26), and *recA*(7). Serotype prediction based on the draft genome using SerotypeFinder. “Oxx:Hyy” indicates additional data using Abricate analysis.

**Table 2:** Overview of draft genomes.

Assembly	Contig	Total Length (bp)	Largest Contig (bp)	N50 (bp)	GC (%)	CDS
0135	157	4,909,247	363,240	110,005	50.83	4567
0190	271	5,238,332	677,836	147,320	50.51	4960
0198	143	5,061,456	433,368	138,030	50.69	4710
0232	233	5,131,532	332,049	154,315	50.62	4877
0320	147	4,655,431	312,439	84,242	50.7	4380
0350	157	5,339,158	355,221	131,256	50.68	4957
0456	84	4,810,807	484,699	199,978	50.79	4465
0492	153	5,189,267	626,531	317,074	50.47	4808
0513	154	5,086,218	338,786	172,951	50.75	4727
0533	130	5,104,287	575,414	212,669	50.69	4747
0549	88	4,856,633	656,256	279,176	50.55	4461

## Data Availability

The original contributions presented in the study are included in the article/Supplementary Material; further inquiries can be directed to the corresponding author.

## Supplementary Materials

The following supporting information can be downloaded at: https://www.mdpi.com/article/10.3390/microorganisms12061139/s1, Table S1: Prediction of serotypes; Table S2: Identification of antibacterial genes using Abricate and the CARD database; Table S3: Identification of antibacterial genes using Abricate and the ResFinder database. Table S4: Information on plasmids harboured by the *E. coli* isolates.
