# Supplementary material for: Genome Sequences of Extended-Spectrum Beta-Lactamase-Producing Escherichia coli Recovered from Mid-Stream Urine Samples in Accra, Ghana
Source: Microorganisms. 2024 Jun 4;12(6):1139. doi: 10.3390/microorganisms12061139 (PMC11205560; doi:10.3390/microorganisms12061139)
Supplement: Supplementary file 1 [file microorganisms-12-01139-s001.zip › microorganisms-3024571-supplementary.pdf]

Table S1: Prediction of serotypes

				Isolate 0190			
**SerotypeFinder**							
Database	Gene	Serotype	Identity	Template / HSP length	Contig	Position in contig	Accession number
O_type	wzy	O96	100	1251 / 1251	Contig_1	614320..615570	AB812043
O_type	wzx	O96	100	1248 / 1248	Contig_1	611015..612262	AB812043
H_type	fliC	H19	99.89	1832 / 1833	Contig_1	768909..770740	AY337479
**Abricate**							
#FILE	GENE	COVERAGE	%COVERAGE	%IDENTITY	DATABASE	ACCESSION	PRODUCT
Ec_0190	fliC-H19	1-1832/1833	99.95	99.89	ecoh	AY337479.1	flagellin H19
Ec_0190	wzx-O96	1-1248/1248	100	100	ecoh	AB812043.1	O antigen flippase O96
Ec_0190	wzy-O96	1-1251/1251	100	100	ecoh	AB812043.1	O antigen polyermase O96
				Isolate 0198			
**SerotypeFinder**							
Database	Gene	Serotype	Identity	Template / HSP length	Contig	Position in contig	Accession number
H_type	fliC	H10	99.84	1263 / 1263	Contig_31	10674..11936	AY249995
H_type	fliC	H10	99.92	1262 / 1263	Contig_31	10675..11936	AY337482
O_type	wzm	O101	100	780 / 780	Contig_58	6631..7410	CP011061
O_type	wzt	O101	100	753 / 753	Contig_58	5872..6624	CP011061
**Abricate**							
#FILE	GENE	COVERAGE	%COVERAGE	%IDENTITY	DATABASE	ACCESSION	PRODUCT
Ec_0198	fliC-H10	1-1262/1263	99.92	99.92	ecoh	AY337482.1	flagellin H10
Ec_0198	wzm-Onovel32	1-753/753	100	100	ecoh	SRR2544773	O-antigen ABC transporter permease Onovel32
Ec_0198	wzt-Onovel32	1-780/780	100	100	ecoh	SRR2544773	ATP-binding protein Onovel32
				Isolate 0232			
**SerotypeFinder**							

Database	Gene	Serotype	Identity	Template / HSP length	Contig	Position in contig	Accession number
H_type	fliC	H12	99.94	1788 / 1788	Contig_6	159610..161397	AIFX01000055
O_type	wzx	O100	100	1211 / 1242	Contig_55	9595..10805	AB812045
O_type	wzy	O100	99.91	1170 / 1170	Contig_55	7212..8381	AB812045
**Abricate**							
#FILE	GENE	COVERAGE	%COVERAGE	%IDENTITY	DATABASE	ACCESSION	PRODUCT
Ec_0232	fliC-H12	1-1788/1788	100	99.83	ecoh	AY337471.1	flagellin H12
Ec_0232	wzx-O100	32-1242/1242	97.5	100	ecoh	AB812045.1	O antigen flippase O100
Ec_0232	wzy-O100	1-1170/1170	100	99.92	ecoh	AB812045.1	O antigen polyermase O100
Isolate 0320							
**SerotypeFinder**							
Database	Gene	Serotype	Identity	Template / HSP length	Contig	Position in contig	Accession number
H_type	fliC	H30	99.94	1713 / 1713	Contig_21	40675..42387	AY250011
O_type	wzm	O62	100	768 / 768	Contig_75	6044..6811	KY379508
O_type	wzx	O68	99.92	1212 / 1212	Contig_9	75184..76395	AB812027
O_type	wzy	O68	99.91	1079 / 1107	Contig_9	72077..73155	AB812027
**Abricate**							
#FILE	GENE	COVERAGE	%COVERAGE	%IDENTITY	DATABASE	ACCESSION	PRODUCT
Ec_0320	fliC-H30	1-1713/1713	100	99.94	ecoh	AY250011.1	flagellin H30
Ec_0320	wzx-O62-Gp14	1-1212/1212	100	100	ecoh	AB812023.1	O antigen flippase O62
Ec_0320	wzy-O62-Gp14	1-1107/1107	100	100	ecoh	AB812023.1	O antigen polyermase O62
Isolate 0456							
**SerotypeFinder**							
Database	Gene	Serotype	Identity	Template / HSP length	Contig	Position in contig	Accession number
H_type	fliC	H8	100	1479 / 1479	Contig_2	402456..403934	AJ865465
NO hits for O type							
**Abricate**							

#FILE	GENE	COVERAGE	%COVERAGE	%IDENTITY	DATABASE	ACCESSION	PRODUCT
Ec_0456	fliC-H8	1-1479/1479	100	100	ecoh	AJ865465.1	flagellin H8
Ec_0456	wzx-O8	1-1251/1251	100	99.76	ecoh	AB811598.1	O antigen flippase O8
Ec_0456	wzy-O8	1-1251/1251	100	100	ecoh	AB811598.1	O antigen polyermase O8
				Isolate 0513			
**SerotypeFinder**							
Database	Gene	Serotype	Identity	Template / HSP length	Contig	Position in contig	Accession number
H_type	fliC	H5	99.16	1311 / 1311	Contig_11	75298..76608	AY249990
O_type	wzy	O16	100	1167 / 1167	Contig_5	25097..26263	AB811601
O_type	wzx	O16	100	1248 / 1248	Contig_5	27374..28621	AB811601
**Abricate**							
#FILE	GENE	COVERAGE	%COVERAGE	%IDENTITY	DATABASE	ACCESSION	PRODUCT
Ec_0513	fliC-H5	1-1311/1311	100	99.16	ecoh	AY249990.1	flagellin H5
Ec_0513	wzx-O16	1-1248/1248	100	100	ecoh	AB811601.1	O antigen flippase O16
Ec_0513	wzy-O16	1-1167/1167	100	100	ecoh	AB811601.1	O antigen polyermase O16
				Isolate 0533			
**SerotypeFinder**							
Database	Gene	Serotype	Identity	Template / HSP length	Contig	Position in contig	Accession number
H_type	fliC	H5	99.16	1311 / 1311	Contig_7	136316..137626	AY249990
O_type	wzy	O16	100	1167 / 1167	Contig_2	25097..26263	AB811601
O_type	wzx	O16	100	1248 / 1248	Contig_2	27374..28621	AB811601
**Abricate**							
#FILE	GENE	COVERAGE	%COVERAGE	%IDENTITY	DATABASE	ACCESSION	PRODUCT
Ec_0533	fliC-H5	1-1311/1311	100	99.16	ecoh	AY249990.1	flagellin H5
Ec_0533	wzx-O16	1-1248/1248	100	100	ecoh	AB811601.1	O antigen flippase O16
Ec_0533	wzy-O16	1-1167/1167	100	100	ecoh	AB811601.1	O antigen polyermase O16
				Isolate 0549			
**SerotypeFinder**							
Database	Gene	Serotype	Identity	Template / HSP length	Contig	Position in contig	Accession number

H_type	fliC	H25	98.03	1320 / 1332	Contig_3	452312..453631	AGSG01000116
O_type	wzx	O1	100	1227 / 1227	Contig_16	44095..45321	GU299791
O_type	wzy	O1	99.7	1005 / 1005	Contig_16	47589..48593	GU299791
**Abricate**							
#FILE	GENE	COVERAGE	%COVERAGE	%IDENTITY	DATABASE	ACCESSION	PRODUCT
Ec_0549	fliC-H25	1-1196/1197	99.92	98.08	ecoh	AY250007.1	flagellin H25
Ec_0549	wzx-O1	1-1227/1227	100	100	ecoh	GU299791.1	O antigen flippase O1
Ec_0549	wzy-O1	1-1005/1005	100	99.7	ecoh	GU299791.1	O antigen polyermase O1
Isolate 0492							
**SerotypeFinder**							
Database	Gene	Serotype	Identity	Template / HSP length	Contig	Position in contig	Accession number
H_type	fliC	H1	99.94	1788 / 1788	Contig_1	239507..241294	AB028471
O_type	wzx	O4	100	1239 / 1239	Contig_2	583341..584579	AY568960
O_type	wzy	O4	100	1188 / 1188	Contig_2	585475..586662	AY568960
**Abricate**							
#FILE	GENE	COVERAGE	%COVERAGE	%IDENTITY	DATABASE	ACCESSION	PRODUCT
Ec_0492	fliC-H1	1-1788/1788	100	99.94	ecoh	AB028471.1	flagellin H1
Ec_0492	wzx-O4	1-1239/1239	100	100	ecoh	AY568960.1	O antigen flippase O4
Ec_0492	wzy-O4	1-1188/1188	100	100	ecoh	AY568960.1	O antigen polyermase O4
Isolate 0135							
**SerotypeFinder**							
Database	Gene	Serotype	Identity	Template / HSP length	Contig	Position in contig	Accession number
H_type	fliC	H4	99.9	1050 / 1050	Contig_1	7267..8316	AJ605764
O_type	wzt	O101	100	753 / 753	Contig_66	3400..4152	CP011061
O_type	wzm	O101	100	780 / 780	Contig_66	4159..4938	CP011061
**Abricate**							
#FILE	GENE	COVERAGE	%COVERAGE	%IDENTITY	DATABASE	ACCESSION	PRODUCT
Ec_0135	fliC-H4	1-1050/1050	100	99.91	ecoh	AJ605764.1	flagellin H4
Ec_0135	wzm-Onovel32	1-753/753	100	100	ecoh	SRR2544773	O-antigen ABC transporter permease Onovel32

Ec_0135	wzt-Onovel32	1-780/780	100	100	ecoh	SRR2544773	ATP-binding protein Onovel32
Isolate 0350							
**SerotypeFinder**							
Database	Gene	Serotype	Identity	Template / HSP length	Contig	Position in contig	Accession number
H_type	fliC	H16	99.94	1575 / 1575	Contig_20	69251..70825	JH953794
O_type	wzy	O45	100	1092 / 1092	Contig_7	171256..172347	CU463050
O_type	wzx	O45	100	1263 / 1263	Contig_7	169037..170299	CU463050
**Abricate**							
#FILE	GENE	COVERAGE	%COVERAGE	%IDENTITY	DATABASE	ACCESSION	PRODUCT
Ec_0350	fliC-H16	1-1570/1578	99.49	99.94	ecoh	AY337475.1	flagellin H16
Ec_0350	wzx-O45	1-1263/1263	100	100	ecoh	CU463050.1	O antigen flippase O45
Ec_0350	wzy-O45	1-1092/1092	100	100	ecoh	CU463050.1	O antigen polyermase O45

Table S2: Identification of antibacterial genes using Abricate and the CARD database

GENE	ACCESSION	PRODUCT	RESISTANCE	Ec-0135	Ec-0190	Ec-0198	Ec-0232	Ec-0320	Ec-0350	Ec-0456	Ec-0492	Ec-0513	Ec-0533	Ec-0549
AAC(3)-IIe	EU022315.1:0-861	AAC(3)-IIe is a plasmid-encoded aminoglycoside acetyltransferase in E. coli	Aminoglycoside	0	0	1	0	0	0	0	0	0	0	0
AAC(6')-Ib-cr	DQ303918:0-600	AAC(6')-Ib-cr is an aminoglycoside acetyltransferase encoded by plasmids transposons integrons in Enterobacteriaceae. The aac(6')-Ib-cr variant gene can induce resistance against aminoglycoside and fluoroquinolone simultaneously	Aminoglycoside; Fluoroquinolone	1	0	1	0	0	0	0	0	0	0	0
aadA5	AF137361:63-852	aadA5 is an aminoglycoside nucleotidyltransferase gene encoded by plasmids transposons and integrons in E. coli K. pneumoniae Kluyvera georgiana P. aeruginosa and E. cloacae	Aminoglycoside	1	0	1	0	0	0	0	0	1	1	0
acrA	U00096.3:485619-484425	AcrA is a subunit of the AcrAB-TolC	Cephalosporin; Fluoroquinolone;	1	1	1	1	1	1	1	1	1	1	1

		multidrug efflux system that in E. coli.	Glycylcycline; Penam; Phenicol; Rifamycin; Tetracycline; Triclosan											
acrB	U00096.3:484403-481253	Protein subunit of AcrA-AcrB-TolC multidrug efflux complex. AcrB functions as a herterotrimer which forms the inner membrane component and is primarily responsible for substrate recognition and energy transduction by acting as a drug/proton antiporter.	Cephalosporin; Fluoroquinolone; Glycylcycline; Penam; Phenicol; Rifamycin; Tetracycline; Triclosan	1	1	1	1	1	1	1	1	1	1	1
acrD	AP009048.1:2586250-2589364	AcrD is an aminoglycoside efflux pump expressed in E. coli. Its expression can be induced by indole and is regulated by baeRS and cpxAR.	Aminoglycoside	1	1	1	1	1	1	1	1	1	1	1
acrE	U00096:3413863-3415021	AcrE is a membrane fusion protein similar to AcrA.	Cephalosporin; Cephamycin; Fluoroquinolone; Penam	1	1	0	1	1	1	1	1	1	1	1
acrF	U00096:3415032-3418137	AcrF is an inner membrane transporter similar to AcrB.	Cephalosporin; Cephamycin; Fluoroquinolone; Penam	1	1	1	1	1	1	1	1	1	1	1

acrS	U00096:3413465-3412802	AcrS is a repressor of the AcrAB efflux complex and is associated with the expression of AcrEF. AcrS is believed to regulate a switch between AcrAB and AcrEF efflux.	Cephalosporin; Cephamycin; Fluoroquinolone; Glycylcycline; Penam; Phenicol; Rifamycin; Tetracycline; Triclosan	1	1	1	1	1	0	1	1	1	1	1
ampC	U00096.3:4378944-4377810	A class C ampC beta-lactamase (cephalosporinase) enzyme described in Escherichia coli shown clinically to confer resistance to penicillin-like and cephalosporin-class antibiotics.	Cephalosporin; Penam	1	1	1	1	1	1	1	1	1	1	1
ampC1	FN649414.1:2765050-2766355	An ampC-like beta-lactamase identified from Escherichia coli.	Cephalosporin; Penam	1	1	1	1	1	1	1	0	0	0	1
ampH	AP012030.1:396711-395553	AmpH is a class C ampC-like beta-lactamase and penicillin-binding protein identified in Escherichia coli.	Cephalosporin; Penam	1	1	1	1	1	1	1	1	1	1	1
APH(3'')-Ib	AF313472:15593-16397	APH(3'')-Ib is an aminoglycoside phosphotransferase encoded by plasmids transposons integrative	Aminoglycoside	1	0	1	1	1	1	1	1	1	1	1

		conjugative elements and chromosomes in Enterobacteriaceae and Pseudomonas spp.												
APH(6)-Id	AF024602:3155-3992	APH(6)-Id is an aminoglycoside phosphotransferase encoded by plasmids integrative conjugative elements and chromosomal genomic islands in K. pneumoniae Salmonella spp. E. coli Shigella flexneri Providencia alcalifaciens Pseudomonas spp. V. cholerae Edwardsiella tarda Pasteurella multocida and Aeromonas bestiarum	Aminoglycoside	1	0	1	1	1	1	1	1	1	1	1
bacA	U00096.3:3204131-3203309	The bacA gene product (BacA) recycles undecaprenyl pyrophosphate during cell wall biosynthesis which confers resistance to bacitracin.	Peptide	1	1	1	1	1	1	1	1	1	1	1

baeR	AP009048.1:2166412-2167135	BaeR is a response regulator that promotes the expression of MdtABC and AcrD efflux complexes.	Aminocoumarin; Aminoglycoside	1	1	1	1	1	1	1	1	1	1	1
baeS	AP009048:2165012-2166416	BaeS is a sensor kinase in the BaeSR regulatory system. While it phosphorylates BaeR to increase its activity BaeS is not necessary for overexpressed BaeR to confer resistance.	Aminocoumarin; Aminoglycoside	1	1	1	1	1	1	1	1	1	1	1
catI	V00622:243-903	catI is a chromosome and transposon-encoded variant of the cat gene found in Escherichia coli and Acinetobacter baumannii	Phenicol	0	0	0	1	0	0	1	1	0	0	0
cpxA	BA000007.3:4905062-4903688	CpxA is a membrane-localized sensor kinase that is activated by envelope stress. It starts a kinase cascade that activates CpxR which promotes efflux complex expression.	Aminocoumarin; Aminoglycoside	1	1	1	1	1	1	0	1	1	1	1

CRP	AP009048.1:4154296-4153663	CRP is a global regulator that represses MdtEF multidrug efflux pump expression.	Fluoroquinolone; Macrolide; Penam	1	1	1	1	1	1	1	1	1	1	1
CTX-M-15	AY044436:1435-2311	CTX-M-15 is a beta-lactamase found in the Enterobacteriaceae family	Cephalosporin	1	1	1	0	1	0	0	0	1	1	1
CTX-M-3	Y10278:0-876	CTX-M-3 is a beta-lactamase found in Citrobacter freundii	Cephalosporin	0	0	0	1	0	0	0	0	0	0	0
dfrA1	KJ541681.1:19526-20000	dfrA1 is an integron-encoded dihydrofolate reductase	Diaminopyrimidine	0	1	0	1	0	0	0	0	0	0	0
dfrA14	KF921535.1:617-1091	dfrA14 is an integron-encoded dihydrofolate reductase found in Escherichia coli	Diaminopyrimidine	0	0	0	0	1	0	1	0	0	0	1
dfrA17	DQ838665:0-474	dfrA17 is an integron-encoded dihydrofolate reductase found in Escherichia coli	Diaminopyrimidine	1	0	1	0	0	0	0	0	1	1	0
dfrA7	FJ854362:2338-2812	dfrA7 is an integron-encoded dihydrofolate reductase found in Escherichia coli	Diaminopyrimidine	0	0	0	0	0	0	0	1	0	0	0
emrA	AP009048:2810082-2811255	EmrA is a membrane fusion protein providing an efflux pathway with EmrB and TolC between the inner and outer	Fluoroquinolone	1	1	1	1	1	1	1	1	1	1	1

		membranes of E. coli a Gram-negative bacterium.												
emrB	U00096:2812615-2814154	emrB is a translocase in the emrB -TolC efflux protein in E. coli. It recognizes substrates including carbonyl cyanide m-chlorophenylhydrazone (CCCP) nalidixic acid and thioloactomycin.	Fluoroquinolone	1	1	1	1	1	1	1	1	1	1	1
emrE	Z11877.1:485-818	Member of the small MDR (multidrug resistance) family of transporters; in Escherichia coli this protein provides resistance against a number of positively charged compounds including ethidium bromide and erythromycin; proton-dependent secondary transporter which exchanges protons for compound translocation	Macrolide	1	1	1	0	1	1	1	1	1	1	1

emrK	D78168:536-1592	emrK is a membrane fusion protein that is a homolog of EmrA. Together with the inner membrane transporter EmrY and the outer membrane channel TolC it mediates multidrug efflux.	Tetracycline	1	1	1	1	1	1	1	1	1	1	1
emrR	U00096.3:2810769-2811300	EmrR is a negative regulator for the EmrAB-TolC multidrug efflux pump in E. coli. Mutations lead to EmrAB-TolC overexpression.	Fluoroquinolone	1	1	1	1	1	1	1	1	1	1	1
emrY	D78168:1591-3130	emrY is a multidrug transport that moves substrates across the inner membrane of the Gram-negative E. coli. It is a homolog of emrB.	Tetracycline	1	1	1	1	1	1	1	1	1	1	1
eptA	AP009048:4340268-4338624	PmrC mediates the modification of Lipid A by the addition of 4-amino-4-deoxy-L-arabinose (L-Ara4N) and phosphoethanolamine resulting in a less negative cell membrane and	Peptide	1	1	1	1	1	1	1	1	1	1	1

		decreased binding of polymyxin B.												
ErmB	AF242872.1:2131-2878	ErmB confers the MLSb phenotype. Similar to ErmC expression of ErmB is inducible by erythromycin. The leader peptide causes attenuation of the mRNA and stabilizes the structure preventing further translation. When erythromycin is present it binds the leader peptide causing a change in conformation allowing for the expression of ErmB.	Lincosamide; Macrolide; Streptogramin	0	0	0	1	0	0	0	0	0	0	0
evgA	BA000007.3:3212025-3212640	EvgA when phosphorylated is a positive regulator for efflux protein complexes emrKY and mdtEF. While usually phosphorylated in a EvgS dependent manner it can be phosphorylated in the absence of	Fluoroquinolone; Macrolide; Penam; Tetracycline	1	1	1	1	1	1	1	0	1	1	1

		EvgS when overexpressed.												
evgS	U00096:2484373-2487967	EvgS is a sensor protein that phosphorylates the regulatory protein EvgA. evgS corresponds to 1 locus in Pseudomonas aeruginosa PAO1 and 1 locus in Pseudomonas aeruginosa LESB58.	Fluoroquinolone; Macrolide; Penam; Tetracycline	1	1	1	1	1	1	1	0	1	1	1
gadW	CP015085.1:2552440-2551711	GadW is an AraC-family regulator that promotes mdtEF expression to confer multidrug resistance. GadW inhibits GadX-dependent activation. GadW clearly represses gadX and in situations where GadX is missing activates gadA and gadBC.	Fluoroquinolone; Macrolide; Penam	1	1	1	1	1	1	1	1	1	1	1
gadX	AP009048.1:3974604-3975429	GadX is an AraC-family regulator that promotes mdtEF expression to confer	Fluoroquinolone; Macrolide; Penam	1	1	1	1	1	1	1	1	1	1	1

		multidrug resistance.												
H-NS	BA000007.3:1738104-1737690	H-NS is a histone-like protein involved in global gene regulation in Gram-negative bacteria. It is a repressor of the membrane fusion protein genes acrE mdtE and emrK as well as nearby genes of many RND-type multidrug exporters.	Cephalosporin; Cephamycin; Fluoroquinolone; Macrolide; Penam; Tetracycline	1	1	1	1	1	1	1	1	1	1	1
kdpE	U00096.3:721733-721055	kdpE is a transcriptional activator that is part of the two-component system KdpD/KdpE that is studied for its regulatory role in potassium transport and has been identified as an adaptive regulator involved in the virulence and intracellular survival of pathogenic bacteria. kdpE regulates a range of virulence loci through direct promoter binding.	Aminoglycoside	1	1	1	1	1	1	1	1	1	1	1

marA	AP009048.1:1621287-1621671	In the presence of antibiotic stress E. coli overexpresses the global activator protein MarA which besides inducing MDR efflux pump AcrAB also down-regulates synthesis of the porin OmpF.	Carbapenem; Cephalosporin; Cephamycin; Fluoroquinolone; Glycylcycline; Monobactam; Penam; Penem; Phenicol; Rifamycin; Tetracycline; Triclosan	1	1	1	1	1	1	1	1	1	1	1
mdfA	JQ394987:0-1233	Multidrug efflux pump in E. coli. This multidrug efflux system was originally identified as the Cmr/CmlA chloramphenicol exporter.	Benzalkonium_Chloride; Rhodamine; Tetracycline	1	1	1	1	1	1	1	1	1	1	1
mdtA	U00096:2154015-2155263	MdtA is the membrane fusion protein of the multidrug efflux complex mdtABC.	Aminocoumarin	1	1	1	1	1	1	1	1	1	1	1
mdtB	U00096:2155262-2158385	MdtB is a transporter that forms a heteromultimer complex with MdtC to form a multidrug transporter. MdtBC is part of the MdtABC-TolC efflux complex.	Aminocoumarin	1	1	1	1	1	1	1	1	1	1	1

mdtC	U00096:2158385-2161463	MdtC is a transporter that forms a heteromultimer complex with MdtB to form a multidrug transporter. MdtBC is part of the MdtABC-TolC efflux complex. In the absence of MdtB MdtC can form a homomultimer complex that results in a functioning efflux complex with a narrower drug specificity. mdtC corresponds to 3 loci in Pseudomonas aeruginosa PAO1 (gene name: muxC/muxB) and 3 loci in Pseudomonas aeruginosa LESB58.	Aminocoumarin	1	1	1	1	1	1	1	1	1	1	1
mdtE	AP009048.1:3981183-3980025	MdtE is the membrane fusion protein of the MdtEF multidrug efflux complex. It shares 70% sequence similarity with AcrA.	Fluoroquinolone; Macrolide; Penam	1	1	1	1	1	1	1	1	1	1	1

mdtF	U00096:3660413-3663527	MdtF is the multidrug inner membrane transporter for the MdtEF-TolC efflux complex.	Fluoroquinolone; Macrolide; Penam	1	1	1	1	1	1	1	1	1	1	1
mdtG	CP000800.1:1192954-1191727	The MdtG protein also named YceE appears to be a member of the major facilitator superfamily of transporters and it has been reported when overexpressed to increase fosfomycin and deoxycholate resistances. mdtG is a member of the marA-soxS-rob regulon.	Fosfomycin	1	1	1	1	1	1	1	1	1	1	1
mdtH	U00096:1125326-1124117	Multidrug resistance protein MdtH	Fluoroquinolone	1	1	1	1	1	1	1	1	1	1	1
mdtM	U00096.3:4568519-4567286	Multidrug resistance protein MdtM	Acridine_Dye; Fluoroquinolone; Lincosamide; Nucleoside; Phenicol	1	1	1	1	1	0	1	1	1	1	1
mdtN	AP009048.1:4307588-4306556	Multidrug resistance efflux pump. Could be involved in resistance to puromycin acriflavine and tetraphenylarsonium chloride.	Acridine_Dye; Nucleoside	1	1	1	1	1	1	1	1	1	1	1

mdtO	AP009048.1:4306557-4304505	Multidrug resistance efflux pump. Could be involved in resistance to puromycin acriflavine and tetraphenylarsonium chloride	Acridine_Dye; Nucleoside	1	1	1	1	1	1	1	1	1	1	1
mdtP	AP009048.1:4304509-4303042	Multidrug resistance efflux pump. Could be involved in resistance to puromycin acriflavine and tetraphenylarsonium chloride	Acridine_Dye; Nucleoside	1	1	1	1	1	1	1	1	1	1	1
mphA	D16251.1:2531-1625	The mphA gene encodes for resistance enzyme MPH(2')-I which preferentially inactivate 14-membered macrolides (e.g.erythromycin telithromycin roxithromycin) over 16-membered macrolides (e.g.tylosin spiramycin). It phosphorylates macrolides at 2'-OH hydroxyl of desosamine sugar of macrolides in a	Macrolide	1	0	1	1	0	0	0	0	1	1	0

		GTP-dependent manner.												
msbA	U00096.3:966620-968369	MsbA is a multidrug resistance transporter homolog from E. coli and belongs to a superfamily of transporters that contain an adenosine triphosphate (ATP) binding cassette (ABC) which is also called a nucleotide-binding domain (NBD). MsbA is a member of the MDR-ABC transporter group by sequence homology. MsbA transports lipid A a major component of the bacterial outer cell membrane and is the only bacterial ABC transporter	Nitroimidazole	1	1	1	1	1	1	1	1	1	1	1

		that is essential for cell viability.												
OXA-1	JN420336.1:2230-1399	OXA-1 is a beta-lactamase found in E. coli	Cephalosporin; Penam	1	0	1	1	0	0	0	0	0	0	0
OXA-181	JN205800:4140-4938	OXA-181 is a beta-lactamase found in Klebsiella pneumoniae	Cephalosporin; Penam	0	0	0	1	0	0	0	0	0	0	0
pmrF	U00096:2367070-2368039	PmrF is required for the synthesis and transfer of 4-amino-4-deoxy-L-arabinose (Ara4N) to Lipid A which allows gram-negative bacteria to resist the antimicrobial activity of cationic antimicrobial peptides and antibiotics such as polymyxin. pmrF corresponds to 1 locus in Pseudomonas aeruginosa PAO1 and 1 locus in Pseudomonas	Peptide	1	1	1	1	1	1	1	1	1	1	1

		aeruginosa LESB58.												
qacEdelta1	U49101.1:1490-1838	QacEdelta1 is a resistance gene conferring resistance to antiseptics. It is different from QacE only at the 3'-terminus.		1	1	1	0	0	0	0	1	1	1	1
QepA2	EU847537.1:1671-3207	QepA2 is a plasmid-mediated quinolone resistance pump found in an Escherichia coli isolate from France	Fluoroquinolone	0	0	0	1	0	0	0	0	0	0	0
QnrS1	DQ485529.1:0-657	QnrS1 is a plasmid-mediated quinolone resistance protein found in Shigella flexneri	Fluoroquinolone	0	1	0	1	1	0	0	0	0	0	0
SAT-1	AB211124:0-525	SAT-2 is a plasmid-mediated streptothricin acetyltransferase which confers resistance to streptothricin a nucleoside antibiotic.	Nucleoside	0	0	0	1	0	0	0	0	0	0	0

		Originally described from an E. coli plasmid sequence by Heim et al. 1989.												
sul1	JF969163:1053-1893	Sul1 is a sulfonamide resistant dihydropteroate synthase of Gram-negative bacteria. It is linked to other resistance genes of class 1 integrons.	Sulfonamide	1	0	1	0	0	0	0	0	1	1	0
sul2	AY055428.1:21084-20268	Sul2 is a sulfonamide resistant dihydropteroate synthase of Gram-negative bacteria usually found on small plasmids.	Sulfonamide	1	1	0	1	1	1	1	1	1	1	1
TEM-181	NG_050218.1:0-1061	TEM-181 is a beta-lactamase.	Cephalosporin; Monobactam; Penam; Penem	0	1	0	1	1	1	1	1	0	0	1
tet(A)	AF534183.1:2970-4245	TetA is a tetracycline efflux pump found in many species of Gram-negative bacteria.	Tetracycline	0	1	0	1	1	1	1	1	1	1	1
tet(B)	AB089595:0-1206	Tet(B) is a tetracycline efflux protein expressed in many Gram-negative bacteria. It confers resistance to	Tetracycline	1	0	1	0	0	0	0	0	0	0	0

		tetracycline doxycycline and minocycline but not tigecycline.												
tolC	FJ768952:0-1488	TolC is a protein subunit of many multidrug efflux complexes in Gram negative bacteria. It is an outer membrane efflux protein and is constitutively open. Regulation of efflux activity is often at its periplasmic entrance by other components of the efflux complex.	Aminocoumarin; Aminoglycoside; Carbapenem; Cephalosporin; Cephamycin; Fluoroquinolone; Glycylcycline; Macrolide; Penam; Penem; Peptide; Phenicol; Rifamycin; Tetracycline; Triclosan	1	1	1	1	1	1	1	1	1	1	1
ugd	U00096:2099613-2098446	PmrE is required for the synthesis and transfer of 4-amino-4-deoxy-L-arabinose (Ara4N) to Lipid A which allows gram-negative bacteria to resist the antimicrobial activity of cationic antimicrobial peptides and antibiotics such as polymyxin	Peptide	0	0	0	0	1	1	0	1	1	1	1

yojI	U00096.3:2308615-2306971	YojI mediates resistance to the peptide antibiotic microcin J25 when it is expressed from a multicopy vector. YojI is capable of pumping out microcin molecules. The outer membrane protein TolC in addition to YojI is required for export of microcin J25 out of the cell. Microcin J25 is thus the first known substrate for YojI.	Peptide	1	1	1	1	1	1	1	1	1	1	1

Table S3: identification of antibacterial genes using Abricate and the ResFInder database

GENE	PRODUCT	RESISTANCE	Ec-0135	Ec-0190	Ec-0198	Ec-0232	Ec-0320	Ec-0350	Ec-0456	Ec-0492	Ec-0513	Ec-0533	Ec-0549
aac(3)-IIa_1	aac(3)-IIa	Gentamicin; Tobramycin	0	0	1	0	0	0	0	0	0	0	0
aac(6')-Ib-cr_1	aac(6')-Ib-cr	Ciprofloxacin	1	0	1	0	0	0	0	0	0	0	0
aadA1_3	aadA1	Spectinomycin; Streptomycin	0	0	0	1	0	0	0	0	0	0	0
aadA5_1	aadA5	Spectinomycin; Streptomycin	1	0	0	0	0	0	0	0	1	1	0
aph(3'')-Ib_5	aph(3'')-Ib	Streptomycin	1	0	1	1	1	1	1	1	1	1	1
aph(6)-Id_1	aph(6)-Id	Streptomycin	1	0	1	1	1	1	1	1	1	1	1
blaCTX-M-15_1	blaCTX-M-15	Amoxicillin; Ampicillin; Aztreonam; Cefepime; Cefotaxime; Ceftazidime; Ceftriaxone; Piperacillin; Ticarcillin	1	1	1	0	1	0	0	0	1	1	1
blaCTX-M-3_1	blaCTX-M-3	Amoxicillin; Ampicillin; Aztreonam; Cefepime; Cefotaxime; Ceftazidime; Ceftriaxone; Piperacillin; Ticarcillin	0	0	0	1	0	0	0	0	0	0	0
blaOXA-1_1	blaOXA-1	Amoxicillin; Amoxicillin+Clavulanic_acid; Ampicillin; Ampicillin+Clavulanic_acid; Cefepime; Piperacillin; Piperacillin+Tazobactam	1	0	1	1	0	0	0	0	0	0	0
blaOXA-181_1	blaOXA-181	Amoxicillin; Amoxicillin+Clavulanic_acid; Ampicillin; Ampicillin+Clavulanic_acid; Cefepime; Ertapenem;	0	0	0	1	0	0	0	0	0	0	0

		Imipenem; Meropenem; Piperacillin; Piperacillin+Tazobactam											
blaTEM-1B_1	blaTEM-1B	Amoxicillin; Ampicillin; Cephalothin; Piperacillin; Ticarcillin	0	1	0	0	1	1	1	1	0	0	1
blaTEM-35_1	blaTEM-35	Amoxicillin; Amoxicillin+Clavulanic_acid; Ampicillin; Ampicillin+Clavulanic_acid; Piperacillin; Piperacillin+Tazobactam; Ticarcillin; Ticarcillin+Clavulanic_acid	0	0	0	1	0	0	0	0	0	0	0
catA1_1	catA1	Chloramphenicol	0	0	0	1	0	0	0	1	0	0	0
dfrA1_10	dfrA1	Trimethoprim	0	1	0	0	0	0	0	0	0	0	0
dfrA1_8	dfrA1	Trimethoprim	0	0	0	1	0	0	0	0	0	0	0
dfrA14_5	dfrA14	Trimethoprim	0	0	0	0	1	0	1	0	0	0	1
dfrA17_1	dfrA17	Trimethoprim	1	0	1	0	0	0	0	0	1	1	0
dfrA7_5	dfrA7	Trimethoprim	0	0	0	0	0	0	0	1	0	0	0
erm(B)_18	erm(B)	Clindamycin; Erythromycin; Lincomycin; Pristinamycin_IA; Quinupristin; Virginiamycin_S	0	0	0	1	0	0	0	0	0	0	0
mdf(A)_1	mdf(A)		1	1	1	1	1	1	1	1	1	1	1
mph(A)_2	mph(A)	Azithromycin; Erythromycin; Spiramycin; Telithromycin	1	0	1	1	0	0	0	0	1	1	0
qepA1_1	qepA1	Ciprofloxacin	0	0	0	1	0	0	0	0	0	0	0
qnrS1_1	qnrS1	Ciprofloxacin	0	1	0	1	1	0	0	0	0	0	1
sul1_5	sul1	Sulfamethoxazole	1	0	1	0	0	0	0	0	1	1	0
sul2_2	sul2	Sulfamethoxazole	1	1	0	1	1	1	1	0	1	1	1
sul2_3	sul2	Sulfamethoxazole	0	0	0	0	0	0	0	1	0	0	0

tet(A)_6	tet(A)	Doxycycline; Tetracycline	0	1	0	1	1	1	1	1	1	1	1
tet(B)_2	tet(B)	Doxycycline; Minocycline; Tetracycline	1	0	1	0	0	0	0	0	0	0	0

Table S4: Information on plasmids harboured by the *E. coli* isolates

ISOLATE	GENE	COVERAGE	%COVERAGE	%IDENTITY	ACCESSION
Ec_0135	Col(MG828)_1	1-262/262	100	92.75	NC_008486
Ec_0135	ColRNAI_1	1-130/130	100	83.97	DQ298019
Ec_0135	ColRNAI_1	1-130/130	100	86.26	DQ298019
Ec_0135	ColRNAI_1	1-118/130	90.77	86.44	DQ298019
Ec_0135	ColRNAI_1	1-130/130	100	85.5	DQ298019
Ec_0135	IncFIA_1	5-388/388	98.97	99.74	AP001918
Ec_0135	IncFIB(AP001918)_1	1-682/682	100	98.39	AP001918
Ec_0135	IncFIC(FII)_1	33-499/499	93.39	83.62	AP001918
Ec_0190	ColRNAI_1	1-130/130	100	88.55	DQ298019
Ec_0190	ColRNAI_1	1-130/130	100	87.79	DQ298019
Ec_0190	IncB/O/K/Z_2	1-152/160	95	96.08	GU256641
Ec_0190	IncB/O/K/Z_2	1-160/160	100	90.74	GU256641
Ec_0190	IncFII_1	1-261/261	100	96.18	AY458016
Ec_0198	ColRNAI_1	1-130/130	100	86.26	DQ298019
Ec_0198	ColRNAI_1	1-130/130	100	87.79	DQ298019
Ec_0198	IncFIA_1	5-388/388	98.97	99.74	AP001918
Ec_0198	IncFIB(AP001918)_1	1-682/682	100	98.39	AP001918
Ec_0198	IncFIC(FII)_1	1-499/499	99.8	84.06	AP001918
Ec_0198	IncI1_1_Alpha	1-142/142	100	100	AP005147
Ec_0232	ColRNAI_1	1-130/130	100	86.26	DQ298019
Ec_0232	IncFIA_1	1-388/388	100	99.74	AP001918
Ec_0232	IncFIB(AP001918)_1	1-682/682	100	97.65	AP001918
Ec_0232	IncFIC(FII)_1	1-499/499	99.8	84.26	AP001918
Ec_0232	IncI1_1_Alpha	1-142/142	100	97.89	AP005147
Ec_0232	IncX3_1	1-374/374	100	100	JN247852
Ec_0232	IncX4_1	1-374/374	100	100	CP002895
Ec_0320	IncY_1	1-765/765	100	99.08	K02380
Ec_0350	IncB/O/K/Z_1	1-151/151	100	100	CU928147
Ec_0350	IncFIA_1	5-388/388	98.97	99.74	AP001918
Ec_0350	IncFIB(AP001918)_1	1-682/682	100	98.39	AP001918
Ec_0350	IncFIC(FII)_1	1-499/499	99.8	84.06	AP001918
Ec_0456	IncHI1B(CIT)_1_pNDM-CIT	1-538/538	100	93.49	JX182975
Ec_0456	p0111_1	1-885/885	100	98.53	AP010962
Ec_0492	ColRNAI_1	1-130/130	100	84.73	DQ298019
Ec_0492	ColRNAI_1	1-130/130	100	87.02	DQ298019
Ec_0492	IncFIB(AP001918)_1	1-682/682	100	99.27	AP001918

Ec_0492	IncFIC(FII)_1	1-499/499	99.6	84.23	AP001918
Ec_0492	IncQ1_1	1-450/450	100	100	HE654726
Ec_0513	Col156_1	13-154/154	92.21	98.59	NC_009781
Ec_0513	IncFIA_1	1-388/388	100	99.74	AP001918
Ec_0513	IncFIB(AP001918)_1	1-682/682	100	99.71	AP001918
Ec_0513	IncFII(pRSB107)_1_pRSB107	1-261/261	100	100	AJ851089
Ec_0513	IncFII(Yp)_1_Yersenia	1-229/230	96.96	88.65	CP000670
Ec_0533	Col156_1	13-154/154	92.21	98.59	NC_009781
Ec_0533	IncFIA_1	1-388/388	100	99.74	AP001918
Ec_0533	IncFIB(AP001918)_1	1-682/682	100	99.71	AP001918
Ec_0533	IncFII(pRSB107)_1_pRSB107	1-261/261	100	100	AJ851089
Ec_0533	IncFII(Yp)_1_Yersenia	1-229/230	96.96	88.65	CP000670
Ec_0549	Col8282_1	1-205/207	99.03	89.76	DQ995353
Ec_0549	ColRNAI_1	1-130/130	100	90.84	DQ298019
Ec_0549	ColRNAI_1	1-130/130	100	90.08	DQ298019
Ec_0549	IncY_1	1-765/765	100	98.82	K02380
